# Non-uptake of viral load testing among people receiving HIV treatment in Gomba district, rural Uganda

**DOI:** 10.1186/s12879-020-05461-1

**Published:** 2020-10-06

**Authors:** Rita Nakalega, Nelson Mukiza, George Kiwanuka, Ronald Makanga-Kakumba, Robert Menge, Hajira Kataike, Joel Maena, Carolyne Akello, Patience Atuhaire, Flavia Matovu-Kiweewa, Cynthia Ndikuno-Kuteesa, Henry Debem, Andrew Mujugira

**Affiliations:** 1 Makerere University-Johns Hopkins University (MU-JHU) Care LTD, Kampala, Uganda; 2 Baylor College of Medicine Children’s Foundation, Kampala, Uganda; 3 School of Public Health, College of Health Sciences, Makerere University, Kampala, Uganda; 4 MRC/UVRI & LSHTM Uganda Research Unit, Entebbe, Uganda; 5 School of Social Sciences, College of Humanities and Social Sciences, Makerere University Kampala, Kampala, Uganda; 6 Department of Public Health and Preventive Medicine, School of Medicine, University of Liverpool, Liverpool, UK; 7 Infectious Diseases Institute, College of Health Sciences, Makerere University, Kampala, Uganda

**Keywords:** HIV, ART, Viral load, Testing, Uganda

## Abstract

**Background:**

Viral load (VL) testing is the gold-standard approach for monitoring human immunodeficiency virus (HIV) treatment success and virologic failure, but uptake is suboptimal in resource-limited and rural settings. We conducted a cross-sectional study of risk factors for non-uptake of VL testing in rural Uganda.

**Methods:**

We conducted a cross-sectional analysis of uptake of VL testing among randomly selected people with HIV (PWH) receiving anti-retroviral treatment (ART) for at least 6 months at all eight primary health centers in Gomba district, rural Uganda. Socio-demographic and clinical data were extracted from medical records for the period January to December 2017. VL testing was routinely performed 6 months after ART initiation and 12 months thereafter for PWH stable on ART. We used descriptive statistics and multivariable logistic regression to evaluate factors associated with non-uptake of VL testing (the primary outcome).

**Results:**

Of 414 PWH, 60% were female, and the median age was 40 years (interquartile range [IQR] 31–48). Most (62.3%) had been on ART > 2 years, and the median duration of treatment was 34 months (IQR 14–55). Thirty three percent did not receive VL testing: 36% of women and 30% of men. Shorter duration of ART (≤2 years) (adjusted odds ratio [AOR] 2.38; 95% CI:1.37–4.12; *p* = 0.002), younger age 16–30 years (AOR 2.74; 95% CI:1.44–5.24; *p* = 0.002) and 31–45 years (AOR 1.92; 95% CI 1.12–3.27; *p* = 0.017), and receipt of ART at Health Center IV (AOR 2.85; 95% CI: 1.78–4.56; *p* < 0.001) were significantly associated with non-uptake of VL testing.

**Conclusions:**

One-in-three PWH on ART missed VL testing in rural Uganda. Strategies to improve coverage of VL testing, such as VL focal persons to flag missed tests, patient education and demand creation for VL testing are needed, particularly for recent ART initiates and younger persons on treatment, in order to attain the third Joint United Nations Program on HIV/AIDS (UNAIDS) 95–95-95 target – virologic suppression for 95% of PWH on ART.

## Introduction

Eastern and Southern Africa is the epicenter of the global HIV epidemic, accounting for 800,000 (47%) of the 1.7 million new HIV infections globally in 2018 [1, 2]. Between 2010 and 2018, the number of new HIV infections in Uganda decreased from 92,000 to 53,000, a 43% reduction [1]. Despite these gains, Uganda is not on track to reach the UNAIDS 95–95-95 targets for HIV epidemic control i.e., 95% of people with HIV (PWH) knowing their HIV status; 95% of people who know their status on treatment; and 95% of people on treatment with suppressed viral loads by 2030 [3]. In 2018, 84% of PWH in Uganda knew their status, 72% were receiving ART and 64% of people on ART were virally suppressed [4]. Viral suppression, i.e., suppression of plasma viral load below the lower limit of detection for commercially available assays (HIV RNA < 50 copies/ml), is the key indicator of HIV treatment success [5–7]. The World Health Organization (WHO) recommends the use of viral load (VL) testing as the gold standard to ensure viral suppression is achieved and sustained [8], but gaps in the VL cascade of care remain [9]. During January–June 2016, the proportion of people on ART ever receiving at least one VL test in seven sub-Saharan African countries was 9–91% [2].

Uganda began scaling up HIV viral load monitoring in 2014, and the proportion of persons on ART receiving VL testing increased from 5% in 2014 to 50% in 2017 [2, 10]. However, nationally representative data on VL testing coverage are scarce among specific populations such as adolescents and young people who have lower prevalence of viral load suppression (42.5%) than adults 50–54 years (74.2%) [11]. In one study of 397 adolescents aged 10–19 years in Uganda, only 238 (60%) had ever received VL testing [11]. Factors limiting uptake of VL testing in resource-limited settings include lack of awareness among providers and patients of the benefits of VL testing, poor adherence to WHO and national guidelines on VL testing, overburdened health care workers, suboptimal training in quality assurance, weak health and laboratory systems, inefficient transport and result delivery systems, limited funding for the scale-up activities, and poor procurement processes [2, 12]. Factors associated with VL uptake in sub-Saharan Africa include older age, longer time on ART and one-stop HIV care models [13, 14].

Few studies have examined patient and health facility-level factors associated with non-uptake of VL testing in rural settings in sub-Saharan Africa [12, 13, 15]. Addressing these factors will inform programmatic implementation of WHO recommendations for universal VL testing, identify specific interventions to support VL monitoring, and help attainment of the third UNAIDS 95–95-95 target [3]. This study aimed to describe factors associated with non-uptake of VL testing among PWH in Gomba district, Uganda.

## Methods

### Subjects and setting

We conducted a cross-sectional study of PWH who received ART between January and December 2017 in Gomba district, rural Uganda. Gomba is one of 136 administrative districts in Uganda (population 160,075; 92% rural; 49% female) [16] with eight primary health care centers providing comprehensive HIV services and ART to approximately 4200 PWH [17]. Uganda’s healthcare system operates on a referral system, in which lower level facilities refer cases to the next level unit. Primary care facilities are organized by administrative division and include Health Center II (parish), Health Center III (sub-county) and Health Center IV (county). Gomba district had one Health Center IV (a mini hospital with surgical and obstetric services managed by a medical doctor) and 7 Health Center III facilities (each with a general outpatient clinic and maternity ward and led by clinical officers) [18]. Following the adoption of HIV ‘test and treat’ policies in 2016, all newly diagnosed PWH are immediately initiated on ART in accordance with Uganda treatment guidelines [19]. VL testing is performed 6 months after initiation of ART and 12 months thereafter for persons stable on ART [19]. Socio-demographic and clinical data are recorded on patient treatment cards (blue card) and PWH are asked to return to clinic every 3 months for adherence assessment and drug refills. At the scheduled visit for VL testing, the date of blood sample collection is documented on the client’s blue card. Samples are sent from ART clinics to a district laboratory hub for transportation to the national reference laboratory. VL results are recorded in viral load registers and blue cards, and provided to PWH at subsequent clinic visits [17].

### Population and procedures

Data were collected from all 8 primary care ART clinics during a 6-week period in mid-October and November 2018. We included all PWH on ART for at least 6 months. We excluded those with unknown duration on ART, including persons transferred-in from other centers. We used the Leslie Kish equation for cross-sectional studies [20] to estimate the sample size of 414 participants, and the proportionate allocation method to determine the number of participants randomly selected from each health facility [21]. Each PWH was given a unique study identifier by facility staff and a list of codes was used to randomly select PWH from each facility. Data were extracted from paper and electronic ART registers and blue cards. The study team reviewed all extracted records to ensure data quality and perform corrections when data errors were identified. The ART start date was used to calculate the duration on ART at the time of the VL test. Dates of blood draw for VL testing, receipt of results at the clinic, and provision of results to PWH were recorded in a Microsoft® Excel database.

### Laboratory methods

HIV testing is performed using serial rapid HIV-tests: Determine® HIV1/2 (Abbott, IL) [screening test], Stat-Pak® HIV1/2 (Chembio Diagnostic Systems, Medford, NY) [confirmatory test], and SDBioline® (Abbott, IL) [tie breaker test] according to national guidelines [22]. Plasma HIV RNA levels are quantified at the Central Public Health Laboratory using the COBASR TaqMan Analyzer or the COBASR AmpliPrep/COBAS TaqMan HIV-1 test, v2.0 (Roche Molecular Diagnostics, South Branchburg, NJ, USA) (17). Turnaround time for VL results is approximately 6–8 weeks.

### Statistical analysis

Non-uptake of VL testing (the primary outcome) was defined as having no VL test performed during the 12-month study period. We used descriptive statistics and a multivariable logistic regression model to assess factors related to non-uptake of VL testing. Baseline factors evaluated included age, sex, marital status, ART duration, and health facility level. Backward elimination, starting with factors with *p* ≤ 0.2 in bivariate analyses, was performed to select a final multivariate model. Goodness of fit was tested using the Hosmer-Lemeshow test [23]. Interaction and confounding were assessed during model building. Two-sided *p*-values of ≤0.05 were considered statistically significant. Statistical analyses were performed using the Statistical Package for the Social Sciences (SPSS) version 20.0 (IBM Corp. Armonk, NY, USA).

## Results

We enrolled a total of 414 PWH, of whom 250 (60%) were female. The median age and duration on ART were 40 years (interquartile range [IQR] 31–48), and 34 months (IQR 14–55), respectively. One-in-three PWH (33%) did not receive VL testing during the study period. Relative to older age (> 46 years), younger age (odds ratio [OR] 2.90; *p* = 0.001 for 16–30 years and OR 1.84; *p* = 0.02 for 31–45 years) were significantly associated with non-uptake of VL testing uptake in univariate analysis (Table 1). Those on ART ≤2 years (OR 2.24; 95% *p* = 0.002) were more likely to have VL testing non-uptake compared to those on ART > 4 years. Similary, receipt of HIV care at Health Center IV level facility (OR 2.27; *p* < 0.001) was also associated with VL testing non-uptake. We observed similar findings in the adjusted model. Younger age remained significantly associated with non-uptake of VL testing (adjusted odds ratio [AOR] 2.74; 95% CI: 1.44–5.24; *p* = 0.002 and AOR 1.92; 95% CI: 1.12–3.27; *p* = 0.017) for 16–30 years and 31–45 years, respectively. ART duration ≤2 years (AOR 2.38; 95% CI: 1.37–4.12; *p* = 0.002) and receipt of ART at Health Center level IV (AOR 2.85; 95% CI: 1.78–4.56; *p* < 0.001) were significantly associated with VL non-uptake.

**Table 1:** Participant characteristics and correlates of VL testing non-uptake

	Frequency (%)	No VL test (%)	Univariate model		Multivariable model	
Characteristic			OR (95% CI)	***p***-value	AOR (95% CI)	***p***-value
Age (years)						
**1–15**	24 (5.8)	6 (25.0)	1.13 (0.41–3.11)	0.81	1.37 (0.48–3.93)	0.55
**16–30**	76 (18.4)	35 (46.1)	2.90 (1.58–5.33)	0.001	2.74 (1.44–5.24)	0.002
**31–45**	182 (44.4)	64 (35.2)	1.84 (1.11–3.07)	0.018	1.92 (1.12–3.27)	0.017
**> 46**	132 (21.0)	30 (22.7)	Referent		Referent	
ART Duration						
**≤ 2 years**	156 (37.7)	67 (42.9)	2.24 (1.35–3.71)	0.002	2.38 (1.37–4.12)	0.002
**> 2–4 years**	127 (30.7)	35 (27.6)	1.31 (0.65–1.97)	0.67	1.35 (0.75–2.44)	0.32
**> 4 years**	131 (31.6)	33 (25.2)	Referent		Referent	
Health Facility						
**Level III**	276 (66.7)	73 (26.4)	Referent		Referent	
**Level IV**	138 (33.3)	62 (44.9)	2.27 (1.48–3.48)	< 0.001	2.85 (1.78–4.56)	< 0.001
Gender						
**Male**	164 (39.6)	59 (36.0)	Referent			
**Female**	250 (60.4)	76 (30.4)	0.77 (0.51–1.18)	0.24		
Marital Status^**a**^						
**Married**	246 (66.5)	81 (32.9)	Referent			
**Unmarried**	124 (33.5)	39 (31.5)	0.94 (0.59–1.49)	0.78		

^a^Data missing for 44 PWH

## Discussion

In this cross-sectional study of 414 PWH receiving ART in rural Uganda, in which the median duration of HIV treatment was approximately 3 years, we found that one-in-three PWH on ART did not receive a VL test during the 12-month study period. Younger age, shorter duration of ART, and receipt of HIV care at a Health Center IV facility were associated with non-uptake of VL testing.

Our finding that 33% of PWH on ART did not receive a VL test in 2017 is in agreement with prior work in South Africa in which 42, 32, and 26% that did not receive VL testing 6 months, 1 year and 2 years, respectively, after starting treatment [24]. Leakages in the VL cascade were observed in Mozambique where non-uptake of a first VL test was 60% in 2015 [13] and in Cameroon where VL non-uptake was 76% in 2017 [25]. Lack of VL testing limits the evaluation of treatment success, i.e., two consecutive VL measurements < 1000 copies/ml [8], and assessment of HIV transmission risk to sexual partners and infants [7, 8]. Prior to scale up of VL testing in Uganda in 2014, only 5% of ART recipients had received VL testing; this proportion was 23% in 2015, 22% in 2016, 50% in 2017, 63% in 2018 and 85% in 2019 [2, 10, 26, 27]. This finding of relatively high non-uptake of VL testing emphasizes the need for governments in resource-limited settings to scale up VL testing in support of “test and treat all” WHO guidelines. However, gaps remain in the VL cascade of care, including missed tests, implementation of enhanced adherence counselling, delays in following up patients with detectable viraemia and delayed switching to second-line treatment after virologic failure is confirmed [9]. Optimizing the VL cascade and expanding coverage requires addressing challenges to VL implementation in resource-poor settings which include poor adherence to WHO and national guidelines on VL testing, lack of awareness of the benefits of VL testing by clinicians and patients, weak health and laboratory systems, low levels of staff training, poor quality assurance leading to sample rejections, high costs for VL consumables, reagents and tests, and lack of civil society mobilization to improve access to VL testing [12]. Inadequate training, lack of knowledge, poor record-keeping, and difficulties with transportation of samples to the central laboratory are barriers to scale-up of VL testing and may have contributed to the non-uptake of VL testing we observed [17, 26]. Strategies to improve uptake of VL testing including point-of-care testing [28], identifying VL focal persons to flag those in need of VL testing, enhanced adherence counseling, patient education, and demand creation for VL testing, are needed to improve coverage of viral suppression and HIV epidemic control in resource-limited settings [29]. A VL champion program increased testing uptake in South Africa from 64 to 90% after 6 months [24]. Quality improvement tools focused on providers and patients improved VL testing uptake in Malawi [26]. Implementation of these strategies may help attain the “third 95” target in Uganda, where VL coverage increased from 10% in 2014 to 85% in 2019 but is still below the target of universal access [26, 27].

We observed significantly higher non-uptake of VL testing among younger PWH. Younger people below age 25 are less likely to receive a VL test relative to those > 25 years [27]. Delivery of HIV services to young people, particularly young men, is challenging even in the setting of well conducted test-and-treat trials [30]. Younger age is associated with lower rates of viral suppression and virologic rebound and higher loss to follow-up [31–33]. However, studies in Myanmar and Zimbabwe did not find associations between age and VL testing uptake [34, 35].

We found that PWH who had been on ART for ≤2 years had a two-fold higher odds of non-uptake of VL testing than those who had been on ART for more than 4 years. Relatively similar findings were found in a retrospective cohort study in Mozambique in which duration of ART ≤12 months was significantly associated with non-uptake of VL testing [13]. Shorter duration on ART is also associated with higher odds of viral non-suppression [12]. To identify patients with unsuppressed VL in need of adherence interventions and at risk of virologic failure, providers should prioritize VL testing after 6 months of ART [36] in accordance with WHO guidelines. Timely targeted adherence counseling decreases the risk of treatment failure and drug resistance, negating the need for more expensive second-line or third-line therapy [37].

The strengths of our cross-sectional study include proportionate allocation methods to randomly select PWH from all eight HIV clinics in Gomba district, and real-world evaluation of VL non-uptake in a rural setting during scale up of VL testing in Uganda. Our study has limitations. Our cross-sectional design did not permit analysis of time-varying covariates, which may influence trends in VL uptake. We retrospectively analyzed routinely collected program data and there were missing data on marital status (10%). Nevertheless, this did not influence study findings. We did not analyse data on education, occupation, household income, and distance to health facilities because they are not routinely collected in public health facilities. Finally, our results are not generalizable beyond rural settings.

In conclusion, we found non-uptake of VL testing in one-third of PWH in rural Uganda. Non-uptake was associated with younger age, shorter duration of ART, and receipt of HIV care at a higher-level primary care facility. Strategies to improve uptake of VL testing, including VL focal persons to flag those in need of VL testing, enhanced adherence counseling, patient education and demand creation for VL testing, are needed to improve coverage of viral suppression and HIV epidemic control in resource-limited settings.

## Data Availability

The dataset used and analyzed during this study is available from the corresponding author on reasonable request.

## Abbreviations

ART: Anti-Retroviral Treatment
HIV: Human immunodeficiency virus
UNAIDS: Joint United Nations Program on HIV/AIDS
PWH: People with HIV
VL: Viral Load
WHO: World Health Organization
